# French cross-cultural adaptation of the Organizational Readiness for Implementing Change (ORIC)

**DOI:** 10.1186/s12913-019-4361-1

**Published:** 2019-07-31

**Authors:** M. Ruest, G. Léonard, A. Thomas, J. Desrosiers, M. Guay

**Affiliations:** 1 Health sciences research programs, Faculty of Medicine and Health Sciences, Université de Sherbrooke, 3001, 12e Avenue Nord, Sherbrooke, Québec J1H 5N4 Canada; 2 Research Centre on Aging, Centre intégré universitaire de santé et de services sociaux (CIUSSS) de l’Estrie - Centre hospitalier universitaire de Sherbrooke (CHUS), 1036, Belvédère Sud, Sherbrooke, Québec J1H 4C4 Canada; 3 School of Rehabilitation, Faculty of Medicine and Health Sciences, Université de Sherbrooke, 3001, 12e Avenue Nord, Sherbrooke, Québec J1H 5N4 Canada; 4 School of Physical and Occupational Therapy, Faculty of Medicine, McGill University, 3654, Promenade Sir-William-Osler, Montréal, Québec H3G 1Y5 Canada; 5 Centre for Medical Education, Faculty of Medicine, McGill University – Lady Meredith House, 1110 Pine Avenue West, Rm 205, Montréal, Québec H3A 1A3 Canada; 6 Faculty of Medicine and Health Sciences, Université de Sherbrooke, 3001, 12e Avenue Nord, Sherbrooke, Québec J1H 5N4 Canada

**Keywords:** Cross-cultural adaptation process, Translation, Organizational readiness, French organizations, Implementation

## Abstract

**Background:**

Organizational readiness is a factor known to influence the predisposition of individuals within an organization to change. Based on Weiner’s organizational theory, the “Organizational Readiness for Implementing Change” (ORIC) questionnaire was developed and validated to measure organizational readiness in healthcare contexts. However, no such tools allow French-speaking organizations to measure this concept. The objectives of this study were to (1) conduct a French cultural adaptation of the ORIC questionnaire, and (2) initiate the study of its psychometric properties.

**Methods:**

(1) Cross-cultural adaptation and translation processes were first conducted with the methodologies of Beaton, Vallerand and Massoubre. (2) Subsequently, internal consistency was documented by calculating Cronbach’s alpha and inter-item and item-to-scale correlations. The study of construct validity was initiated with a confirmatory factor analysis.

**Results:**

A French 10-item scale named the *Réceptivité organisationnelle à l’implantation d’un changement* (*ROIC*) was developed and pretested by 125 occupational therapists working in Quebec homecare services. Cronbach’s alpha values for the 2 item subscales show satisfactory internal consistency (*Commitment*: α = 0.84 and *Efficacy*: α = 0.86). Inter-item correlations revealed that the *ROIC*’s items are moderately related to each other while item-to-total scale correlations pinpoint items that accounts for variance and influence internal consistency. Confirmatory factor analysis allowed the initiation of a substantial documentation of *ROIC*’s model fit with the original version (CFI = 0.89, TLI = 0.85, SRMR = 0.08, and RMSEA = 0.12).

**Conclusions:**

The *ROIC* is a new theory-based and translated questionnaire that can be used to rigorously document the organizational readiness of French organizations. The *ROIC* has the potential to support members of different organizations in the identification of subsequent efforts for the implementation of a change.

**Electronic supplementary material:**

The online version of this article (10.1186/s12913-019-4361-1) contains supplementary material, which is available to authorized users.

## Background

Contextual characteristics are well-recognized for their potential to significantly influence the implementation of interventions [1–3]. Among these characteristics, organizational readiness has been studied because of its critical impact on the subsequent stages of the change process [4–7]. Some authors have identified organizational readiness as one of the strongest predictors/facilitators for the adoption of new practices, policies and programs [8, 9].

Armenakis et al. (1993) initially suggested that the assessment of individuals’ readiness to change in an organization is a good opportunity to gain insight into their beliefs and attitudes regarding the necessity (or not) of implementing a change, and into the organization’s capacity to implement it with success [10]. The concept of organizational readiness covers characteristics related to individuals (e.g., motivation, commitment, self-efficacy) that are well-recognized for their influence on implementation of a change [11–13]. According to Weiner’s organizational theory, organizational readiness is defined as the extent to which members of an organization are psychologically and behaviorally prepared to implement a change in their setting [14]. These definitions highlight the overlap between the organizational and individual characteristics underlying the multilevel aspect of organizational readiness. An approach that considers both contextual- and individual-specific assessments of readiness is highly recommended when planning and conducting knowledge translation (KT) efforts aimed at optimizing the benefits of the applied change [11, 15–17]. Organizational readiness can therefore allow to document the interplay of these characteristics in a KT process.

The study of organizational determinants related to the KT process and strategies used to enhance organizational readiness has been initiated in the scientific literature [17, 18]. However, faced with the paucity of validated tools to measure an organization’s readiness for change [9, 19], particularly within a KT perspective in healthcare contexts [4], Shea and colleagues (2014) developed and validated the “Organizational Readiness for Implementing Change” (ORIC) questionnaire [5]. The ORIC is based on Weiner’s organizational theory (2009), and describes two main facets of organizational readiness: (1) change commitment and (2) change efficacy [14]. The ORIC aims to document an organization’s level of readiness to implement change as perceived by its members, in order to guide them in the identification of strategies and resources relevant to the context [5]. The ORIC is comprised of 10 items divided into two main subscales: (1) five items on change commitment (i.e., do the intended members of the organization want the change?) and (2) five items on change efficacy (i.e., are the intended members of the organization able to change?). Each of these items is scored using a 5-point Likert scale ranging from “Disagree” to “Agree”. Studies of its psychometric properties support its content validity (i.e., content adequacy); construct validity (i.e., factor structure with exploratory and confirmatory factor analysis); and reliability (i.e., inter-rater reliability and inter-rater agreement) in both laboratory and organizational contexts. For example, the reliability of the original version of the ORIC has been documented to inform the variability of perception from an individual- and group-level perspective [5]. The initial 12-item version of the ORIC was revised following the psychometric research reported by Shea et al. (2014). Two items related to the *Efficacy* subscale were removed because of their interpretation about motivation, a concept related to the *Commitment* subscale according to the participants’ perspective. The 10-item version of the ORIC subsequently became the recommended English version, as it only contains the items that most strongly correspond to the two components initially defined for the measurement of organizational readiness [5].

Studies from different health sectors (e.g., pain management, pharmacological and dental care) have since used the 10-item version of the ORIC to document the perception of readiness among members of an organization [20–22]. For example, Sanders et al. (2017) found it useful to use the ORIC to identify healthcare students’ perceptions and concerns about an immersion program in direct patient care and to guide qualitative data collection in mixed methods research that focuses on the underlying concepts of organizational readiness [22]. However, to our knowledge, the French tools documenting organizational characteristics are not based on the underlying conceptualization described above. Rapid and valid screening of organizational readiness should be extended to French organizations to support the identification of their members’ readiness regarding the implementation of a change, thus maximize their chances of success. As such, the aims of this study were to (1) translate and culturally adapt the ORIC questionnaire in French for Canadian organizations, and (2) initiate the study of its psychometric properties.

### Operational model and context of the study

The cross-cultural adaptation process of the ORIC took place in the context of a larger KT study that aimed to analyze the KT process of a clinical algorithm disseminated in Quebec (Canada) homecare services. The evidence-based algorithm, called “Algo”, was devised to support occupational therapists and non-occupational therapists (e.g., home health aides) in using skill mix during the process of allocating bathing equipment to patients struggling with hygiene care [23]. Algo was developed within an integrated KT approach to facilitate its utilization by stakeholders of the healthcare system. The operational model “integrated-Promoting Action on Research Implementation in Health Services” (i-*PARIHS*) was used to document the characteristics related to its utilization among different individuals and contexts [11]. According to the i-*PARIHS* operational model, the barriers and facilitators to a KT process can be associated with either individual characteristics, context, or innovation [11]. In this perspective, organizational readiness can help document both the individual characteristics and the contextual climate present within the organizations. The ORIC therefore has the potential to support the identification of barriers and facilitators related to the members of an organization that are involved in the implementation of a change.

## Methods

A cross-cultural adaptation process of the English version of the ORIC developed by Shea et al. (2014) [5] was conducted based on the six steps of Beaton’s methodology [24] and complemented by Vallerand procedures [25]. In addition, Massoubre et al.’s (2002) procedure was used following the expert committee review to help document the different types of equivalences (i.e., semantical, technical, idiomatic, related to experience, and conceptual) [26].

### Cross-cultural adaptation procedure

#### Step 1: Forward translation

The first step of the process consists of a forward translation of the ORIC from the source language (i.e., English) to the target language (i.e., French). As recommended by Beaton et al. (2000) and Vallerand (1989), two independent bilingual translators (i.e., Forward1 and Forward2) each completed a first forward translation of the ORIC into their mother tongue (i.e., French). Translators Forward1 and Forward2 had different backgrounds (i.e., Forward1: Master’s in Urban Planning with statistical expertise and Forward2: bachelor’s degree in Translation), as well as different levels of professional experience (Forward1: 21 years and Forward2: 3 years).

#### Step 2: Synthesis of the forward translation

This step involved synthesizing the two French versions of the ORIC produced by the translators (Forward1 and Forward2) into one common preliminary version. A conference call moderated by the first author allowed Forward1 and Forward2 to discuss each translation difference. Consensus between the two translators was reached for most of the differences. For the remaining differences, the research team considered the interpretation of the translators and consulted the French scientific literature in the field of organizational change to reach a consensus regarding the final wording.

#### Step 3: Back translation

As recommended by Beaton et al. (2000) and Vallerand (1989), two backwards translations were then completed by two other independent translators (Back1 and Back2). The independence between the translators from steps 1 and 3 helped to rigorously document the adequacy of the chosen terms for the formulation used in the original version of the questionnaire [25]. Contrary to step 1 (*Forward translation*), the translators selected for step 3 were required to have English as their mother tongue, and to have no expertise in the field covered by the questionnaire (i.e., organizational readiness). Back1 and Back2 translators had similar profiles (i.e., bachelor’s degree in Translation) and similar levels of professional experience (i.e., 25 years each). A first pre-final French version of the ORIC, based on the Forward1, Forward2, Back1 and Back2 versions of the questionnaire, was produced in preparation for an expert committee meeting.

#### Step 4: Expert committee review

The expert committee members (*n* = 5) invited to review the French version of the ORIC included (a) three researchers participating in this study (including the third author, who has extensive experience in the field of translating tools [MR; MG; JD]); (b) a specialized translator in the field of healthcare (not involved in previous steps of the translation nor in the research process [MG]); and c) a psychologist with expertise in work organization (field covered by the ORIC [CD]). The committee composition was therefore compatible with the first type (i.e., researchers only) described by Vallerand (1989). The meeting was recorded for further data analysis.

#### Step 5: Pretesting of the French version of the ORIC

As previously specified, the cross-cultural adaptation process of the ORIC aimed to further document the organizational readiness related to a larger study (i.e., analysis of the KT process of a clinical algorithm deployed in Quebec homecare services since 2013) conducted with French Canadian occupational therapists. Prior to the pretest, the pre-final French version of the ORIC was tested with a convenience sample of 9 French-speaking Canadians (i.e., 2 healthcare managers and 7 occupational therapists) using the “think aloud technique” [27]. The “think aloud technique” highlighted the need to define the phrase “People who work here […]” used at the beginning of each item, when completing the questionnaire. Items were otherwise well understood by participants.

For the pretest of the French version of the ORIC (step 5), the target population was defined as occupational therapists who were members of the *Ordre des ergothérapeutes du Québec* (provincial regulatory body) working in homecare services (*n* = 886). Since 99 of them had not consented to be part of research studies or provided an e-mail address where they could be contacted, a sample of 787 occupational therapists received an e-mail invitation on September 25th, 2015 containing the link to the electronic platform *LimeSurvey* (reminder on February 4th, 2016) where the French version of the ORIC was presented. The survey was active for as long as participants continued to answer (data were collected up to March 24th, 2016). No exclusion criteria were considered for this cross-cultural adaptation process. Informed consent was obtained from each participant prior to data collection, on the first page of the survey platform (ethics approval MP-22-2016-532). The respondents had the opportunity to add comments below each item of the French version of the ORIC (e.g., to report unclear sentences).

#### Step 6: Submission and appraisal of reports by committee

Throughout the French cross-cultural adaptation process of the ORIC, the authors communicated by e-mail with the authors of the original English version. These exchanges led us to select the 10-item version of the questionnaire for the cross-cultural adaptation process in French, as per their recommendation, and allowed us to obtain clarifications when needed (e.g., meaning of a word).

### Data analysis

For the cross-cultural adaptation process, all translation results from steps 1 (*Forward translation*) and 3 (*Back translation*) were grouped into tables to facilitate comparison and discussion in steps 2 (*Synthesis of translation*) and 4 (*Expert committee review*). Following the completion of step 4, recorded exchanges between the experts were transcribed and transferred to the NVivo 10 platform (QSR International Pty Ltd; Australia). Each transcript was analyzed and coded according to the type of equivalence (i.e., semantical, technical, idiomatic, related to experience, and/or conceptual) to which it referred, as per the classification of Massoubre et al. (2002) [26].

Descriptive statistics were first used for the characteristics of the respondents in step 5 (*Pretest*). A chi-square for one sample was calculated for the two variables for which information was available from the professional regulatory board: (1) the gender and (2) the administrative region of professional practice. For the second variable, 95% confidence intervals (normal approximation) were calculated for each frequency in order to identify the proportion(s) of the sample that were not representative of the population. Item-level descriptive statistics were also calculated to identify missing and out-of-range data [28]. The internal consistency (i.e., degree of homogeneity) for each of the two subscales (5 items per subscale) of the French version of the ORIC was determined using Cronbach’s alpha coefficient (α). The interpretation cut-off scores of DeVellis et al. (2003) were used to appreciate the degree of homogeneity (i.e., 0.70 ≥ α ≤ 0.90). Inter-item correlations were calculated to ensure a minimal association between items [29]. An average value situated between 0.15 and 0.50 was considered “good” to ensure items were not redundant but still related to the overall concept measured by the questionnaire [30]. Parallel to these calculations, item-to-scale correlations of the French version of the ORIC were conducted to verify each item’s adequacy in individually contributing to the homogenous measurement of the construct of organizational readiness, while maintaining sufficient variance [31].

Finally, a confirmatory factor analysis was conducted with the SAS software 9.4 (CALIS procedure) to initiate the documentation of the French version of the ORIC’s construct validity. The adequacy of its theoretical structure with regards to the items related to the *Commitment* and *Efficacy* facets was estimated with the “Robust maximum likelihood method” (chosen because of the ordinal data). The assessment of the model fit with pretest data (step 5) was completed with the convention used by Shea et al. (2014) for interpreting factor loadings (> 0.60), as well as with four adjustment measures (two incremental and two absolute fit indexes): (1) the comparative fit index (CFI) (≥ 0.95); (2) the Tucker-Lewis index (TLI) (≥ 0.95); (3) the standardized root mean square residual (SRMR) (< 0.08); and (4) the root mean square error of approximation (RMSEA) (< 0.06), as recommended by Hu & Bentler (1999) [32]. The CFI compared the sample covariance matrix with the null (independence) model, while the TLI allowed for the verification of discrepancies between the chi-squared (χ^2^) value of the sample model and the null model. The SRMR was calculated to document the difference of the standardized residuals between the French version of the ORIC’s observed covariance matrix and the ORIC’s hypothesized covariance matrix. Finally, the RMSEA allowed for the verification of discrepancies between the French version of the ORIC’s observed covariance matrix and the hypothesized covariance matrix of the original ORIC.

## Results

### Réceptivité organisationnelle à l’implantation d’un changement (ROIC), the French version of the ORIC

For the first step of Beaton’s process (*Forward Translation*), 20 differences between translators Forward1 and Forward2 were identified. From the preliminary French version of the ORIC obtained at the end of step 2 (*Synthesis of the translation*), the back translation of the questionnaire (step 3) allowed for the identification of 25 differences with the original version of the ORIC, including 11 common to both translators. The differences identified during steps 1 and 3 raised questions about equivalence regarding semantic structure (e.g., translation of the expression “manage the politics”) and technical (e.g., translation of the gerund “implementing”) aspects of certain items (see Table 1).

**Table 1:** Cross-cultural adaptation process of the *Réceptivité organisationnelle à l’implantation d’un changement*

Original version of the ORIC	Step 1: Forward Translation		Step 2: Synthesis - Forward Translation	Step 3: Back Translation		Step 4: Expert committee	Step 5: Pre-test French version of the ORIC	Main equivalences
	*Forward1*	*Forward2*		*Back1*	*Back2*			
Translation differences of the title								
Organizational Readiness for Implementing Change	Préparation organisationnelle à la mise en œuvre d’un changement	Réceptivité de l’organisation à l’implantation d’un changement	Réceptivité organisationnelle à l’implantation d’un changement	Organizational receptivity to the implementation of change	Organizational readiness for implementing change	Réceptivité organisationnelle à l’implantation d’un changement	Réceptivité organisationnelle à l’implantation d’un changement	Conceptual
Translation differences common to several items								
1. People 2. Feel confident 3. Organization 4. Implementing	Personnes Estiment Organisation Mettre en œuvre	Gens Ont confiance Entreprise Implanter	Personnes Estiment Organisation Implanter	Those Believe Organization Implementation Implementing	The people Think Organization Implementing	Les personnes Sont confiantes Organisation Mettre en œuvre		Conceptual Conceptual/technical Conceptual Conceptual/technical
Items of the Organizational Readiness for Implementing Change								
1. People who work here are committed to implementing this change.	Les personnes qui travaillent ici **se sont engagées** à mettre en œuvre ce changement.	Les gens qui travaillent ici **s’investissent** pour implanter ce changement.	Les personnes qui travaillent ici s’engagent à implanter ce changement.	Those who work here are **dedicated** to implementing this change.	2. The people who work here are **committed** to implementing this change.	Les personnes qui travaillent ici s’engagent à mettre en œuvre ce changement.	Les personnes qui travaillent ici s’engagent à mettre en œuvre un changement de pratique clinique.	Conceptual Technical
2. People who work here will do whatever it takes to implement this change.	Les personnes qui travaillent ici **prendront tous les moyens nécessaires** pour mettre en œuvre ce changement.	Les gens qui travaillent ici vont **faire tout ce qui est nécessaire** pour implanter ce changement.	Les personnes qui travaillent ici feront tout ce qui est. nécessaire pour implanter ce changement.	Those who work here will do **everything that is required** to implement this change.	The people who work here will do **everything it takes** to implement this change.	Les personnes qui travaillent ici feront tout ce qui est nécessaire pour mettre en œuvre ce changement.	Les personnes qui travaillent ici feront tout ce qui est nécessaire pour mettre en œuvre un changement de pratique clinique.	Conceptual Technical Semantic
3. People who work here want to implement this change.	Les personnes qui travaillent ici veulent mettre en œuvre ce changement.	Les gens qui travaillent ici veulent implanter ce changement.	Les personnes qui travaillent ici veulent implanter ce changement.	Those who work here want to implement this change.	The people who work here want to implement this change.	Les personnes qui travaillent ici veulent mettre en œuvre ce changement.	Les personnes qui travaillent ici veulent mettre en œuvre un changement de pratique clinique.	
4. People who work here are determined to implement this change.	Les personnes qui travaillent ici sont déterminées à mettre en œuvre ce changement.	Les gens qui travaillent ici sont déterminés à implanter ce changement.	Les personnes qui travaillent ici sont déterminées à implanter ce changement.	Those who work here are determined to implement this change.	The people who work here are determined to implement this change.	Les personnes qui travaillent ici sont déterminées à mettre en œuvre ce changement.	Les personnes qui travaillent ici sont déterminées à mettre en œuvre un changement de pratique clinique.	
5. People who work here are motivated to implement this change.	Les personnes qui travaillent ici sont motivées à mettre en œuvre ce changement.	Les gens qui travaillent ici sont motivés à implanter ce changement.	Les personnes qui travaillent ici sont motivées à implanter ce changement.	Those who work here are motivated to implement this change.	The people who work here are motivated to implement this change.	Les personnes qui travaillent ici sont motivées à mettre en œuvre un changement.	Les personnes qui travaillent ici sont motivées à mettre en œuvre un changement de pratique clinique.	
6. People who work here feel confident that they can handle the challenges that might arise in implementing this change.	Les personnes qui travaillent ici estiment **être en mesure de relever** les défis **que pourrait poser** la mise en œuvre de ce changement.	Les gens qui travaillent ici sont confiants **en leur capacité à bien gérer** les défis **qui peuvent survenir** lors de l’implantation de ce changement.	Les personnes qui travaillent ici estiment pouvoir relever les défis que pourrait poser l’implantation de ce changement.	Those who work here believe that **they can take on** the challenges that this change **could present**.	The people who work here think **they can meet** the challenges that implementing this change **could present**.	Les personnes qui travaillent ici sont confiantes de pouvoir relever d’éventuels défis liés à la mise en œuvre de ce changement.	Les personnes qui travaillent ici sont confiantes de pouvoir relever d’éventuels défis liés à la mise en œuvre d’un changement de pratique clinique.	Conceptual Related to experience Technical
7. People who work here feel confident that they can keep track of progress in implementing this change.	Les personnes qui travaillent ici estiment **être en mesure de suivre l’avancement** de la mise en œuvre de ce changement.	Les gens qui travaillent ici savent **qu’ils peuvent suivre les progrès** de l’implantation de ce changement.	Les personnes qui travaillent ici estiment pouvoir suivre l’avancement de l’implantation de ce changement.	Those who work here believe that **they will be able to follow the advancement** of this implementation of change.	The people who work here think **they can keep keep track of the progress** of implementing this change.	Les personnes qui travaillent ici sont confiantes de pouvoir suivre l’avancement de la mise en œuvre de ce changement.	Les personnes qui travaillent ici sont confiantes de pouvoir suivre l’avancement de la mise en œuvre d’un changement de pratique clinique.	Conceptual Related to experience Technical
8. People who work here feel confident that they can coordinate tasks so that implementation goes smoothly.	Les personnes qui travaillent ici estiment être en mesure de coordonner les tâches **pour assurer** une mise en œuvre **sans heurts**.	Les gens qui travaillent ici ont confiance en leur capacité à bien coordonner les tâches **reliées à** l’implantation pour **qu’elle se passe bien**.	Les personnes qui travaillent ici estiment pouvoir coordonner les tâches pour que l’implantation se passe bien.	Those who work here believe that they can coordinate tasks to **ensure** that the implementation process goes **smoothly**.	The people who work here think they can coordinate the tasks **so that** the implementation goes **well**.	Les personnes qui travaillent ici sont confiantes de pouvoir coordonner les tâches afin que la mise en œuvre se passe bien.	Les personnes qui travaillent ici sont confiantes de pouvoir coordonner les tâches afin que la mise en œuvre d’un changement de pratique clinique se passe bien.	Related to experience Technical
9. People who work here feel confident that the organization can support people as they adjust to this change.	Les personnes qui travaillent ici estiment que l’organisation est. en mesure d’aider les gens à **s’adapter** à ce changement.	Les gens qui travaillent ici ont confiance en l’entreprise pour soutenir les gens **pendant la période d’adaptation** à ce changement.	Les personnes qui travaillent ici estiment que l’organisation peut soutenir les gens pendant la période d’adaptation à ce changement.	Those who work here believe that the organization can **offer** support to the people **as they adapt** to this change.	The people who work here think the organization can support people **while adjusting** to this change.	Les personnes qui travaillent ici sont confiantes que l’organisation peut les soutenir en cours d’adaptation à ce changement.	Les personnes qui travaillent ici sont confiantes que l’organisation peut les soutenir en cours d’adaptation au changement de pratique clinique.	Conceptual Technical
10. People who work here feel confident that they can manage the politics of implementing this change.	Les personnes qui travaillent ici estiment être en mesure de **maîtriser les rouages** de la mise en œuvre de ce changement.	Les gens qui travaillent ici ont confiance en **leur capacité à bien gérer les politiques** en lien avec l’implantation de ce changement.	Les personnes qui travaillent ici estiment pouvoir coordonner les tâches pour que l’implantation se passe bien.	Those who work here believe that they can manage **the policies and implementation procedures** related to this change.	The people who work here think they can manage **the policies and procedures** for implementing this change.	Les personnes qui travaillent ici sont confiantes de pouvoir gérer les enjeux de pouvoir et de reconnaissance au sein du groupe durant la mise en œuvre d’un changement.	Les personnes qui travaillent ici sont confiantes de pouvoir gérer les enjeux de pouvoir et de reconnaissance au sein du groupe durant la mise en œuvre d’un changement de pratique clinique.	Conceptual Related to experience Technical

Using the English and French versions of the questionnaire, step 4 (*Expert committee review*) allowed the research team to discuss each translation difference, item per item, in terms of equivalence. Consensus was achieved for all 10 items following a 90-min meeting with the committee of experts. For the 20 translation differences noted in steps 1 and 3 of the process, an agreement was reached, including for the title wording of the French version of the ORIC. Because the translation differences noted for items 3, 4 and 5 were common to those previously discussed for the other items, these statements were not discussed in detail during the meeting. To this end, the verb “mettre en oeuvre” (as opposed to “implanter”) was adopted to standardize the formulation across items.

Finally, the specifications obtained from the ORIC’s authors throughout the cross-cultural adaptation process for step 6 (*Submission and appraisal of reports by committee*) allowed the team to conduct an in-depth analysis of the differences found between the versions created during steps 1 and 3, and to reach a consensus during the meeting with the expert review committee (step 4). Following the pretest of the French pre-final version of the ORIC (step 5), the specification “[…] de pratique clinique” was added at the end of each item to better contextualize the questionnaire for the solicited population (i.e., occupational therapists). However, to maintain the applicability of the *ROIC* throughout a diversity of professional contexts, this specification was not kept in the final version. In parallel with the pretest of the French pre-final version of the ORIC (step 5), exchanges with the ORIC’s authors at step 6 allowed for the clarification of the wording of two terms: people (items 1–10) and politics (item 10). For instance, the ORIC’s authors specified that the term “politics” refers to the struggles (e.g., activities that aim at improving someone’s status or position) over power between members of the organization during the implementation of a change.

This allowed the final French 10-item version of the *ROIC* to define the *Commitment* (items 1–5) and *Efficacy* (items 6–10) subscales (see Additional file 1).

### Psychometric properties of the ROIC

#### Participant characteristics

Of the 886 occupational therapists working in homecare services, 787 occupational therapists who had agreed to be solicited for research purposes were contacted by e-mail to request their participation in the documentation of the psychometric properties of the *ROIC*. Among the 470 occupational therapists reached, 125 completed the final version (participation rate: 16%). The sample, comprised of 118 women (94%), was representative of Quebec occupational therapists’ gender distribution (92%; *p* > 0.05) as reported by the professional regulatory board (2015–2016). Participants had been working as occupational therapists for 15.3 years [0.5–36] on average, and in Quebec homecare services (public funded health system) for 9.9 years [0.5–29] on average. Respondent distribution across the province was statistically different (*p* < 0.05) from that identified by the occupational therapists’ professional regulatory board at the end of the survey period. This difference mainly arose from the absence of respondents in the “Mauricie-et-Centre-du-Québec” administrative region (see Table 2).

**Table 2:** Sociodemographic characteristics of participants

Variables	Occupational therapists participants of the study (*n* = 125)		Population of Quebec occupational therapists (*n* = 4922)	
	n	% [95% confidence intervals]	N	%
Sociodemographic characteristics				
Gender				
Female	118	94.4	4534	92.1
Male	7	5.6	388	7.9
Highest level of education				
Bachelor’s degree in occupational therapy	91	72.8		
Professional Master’s degree in occupational therapy	24	19.2		
Research Master’s degree	6	4.8		
Other	4	3.2		
Professional experience of participants				
As an occupational therapist, years				
0–10	41	32.8		
11–20	45	36.0		
21–30	31	24.8		
31–40	8	6.4		
As an occupational therapist working in homecare services, years				
0–10	73	58.4		
11–20	39	31.2		
21–30	13	10.4		
Quebec administrative region of professional practice				
1. Bas-St-Laurent	5	4.0 [0.6; 7.4]	115	2.3
2. Saguenay – Lac-St-Jean	9	7.2 [2.7; 11.7]	141	2.8
3. Capitale Nationale	10	8.0 [3.2; 12.8]	628	12.5
4. Mauricie-et-Centre-du-Québec	0	0	336	6.7
5. Estrie	11	8.8 [3.8; 13.8]	227	6.7
6. Montréal	30	24.0 [16.5; 31.5]	1519	30.2
7. Outaouais	8	6.4 [2.1; 10.7]	179	3.6
8. Abitibi-Témiscamingue	2	1.6 [0; 3.8]	57	1.1
9. Côte-Nord	1	0.8 [0; 2.4]	39	0.8
10. Nord-du-Québec	1	0.8 [0; 2.4]	17	0.3
11. Gaspésie – Îles-de-la-Madeleine	3	2.4 [0; 5.1]	46	0.9
12. Chaudière-Appalaches	11	8.8 [3.8; 13.8]	230	4.6
13. Laval	5	4.0 [0.6; 7.4]	237	4.7
14. Lanaudière	6	4.8 [1.1; 8.5]	231	4.6
15. Laurentides	8	6.4 [2.1; 10.7]	282	5.6
16. Montérégie	15	12.0 [6.3; 17.7]	742	14.8

#### Internal consistency of the *ROIC*’s items

The Cronbach alpha values for the *ROIC* items demonstrated satisfactory internal consistency for both item subscales, with α = 0.84 for the *Commitment* subscale and α = 0.86 for the *Efficacy* subscale, as well as for the overall questionnaire (α = 0.91) [29]. The inter-item correlation coefficients of the French version of the ORIC ranged from 0.32 to 0.73, except for the correlation between items 3 and 9 (r = 0.21; see Table 3). When divided into their respective subscales, the inter-item correlation coefficients of the *Commitment* and *Efficacy* subscales ranged from 0.32 to 0.73 and from 0.45 to 0.64 respectively. When grouped by subscale, the item-to-total scale correlation coefficients varied from 0.53 to 0.76 for the *Commitment* subscale, and from 0.63 to 0.72 for the *Efficacy* subscale. Specifically, item 1 tended to explain a lower proportion of variance (0.34) than the other items of the *Commitment* subscale. The Cronbach’s alpha coefficients of the *Commitment* and *Efficacy* subscales ranged from 0.78 to 0.84 and from 0.82 to 0.84 respectively (see Table 4). When grouped together (i.e., item-to-total scale statistics of the 10-item version of the *ROIC)*, the corrected correlation coefficients ranged from 0.62 to 0.78, except for items 3 (r = 0.54) and 9 (r = 0.58). Variation in Cronbach’s alpha coefficients removed oscillated between 0.89 and 0.91 (see Table 5).

**Table 3:** Inter-item correlation matrix of the *Réceptivité organisationnelle à l’implantation d’un changement*

*ROIC* items	Item 1	Item 2	Item 3	Item 4	Item 5	Item 6	Item 7	Item 8	Item 9	Item 10
Item 1	1.00									
Item 2	0.54	1.00								
Item 3	0.32	0.34	1.00							
Item 4	0.45	0.49	0.63	1.00						
Item 5	0.44	0.51	0.64	0.73	1.00					
Item 6	0.51	0.47	0.48	0.68	0.70	1.00				
Item 7	0.63	0.57	0.32	0.44	0.52	0.64	1.00			
Item 8	0.35	0.44	0.37	0.49	0.60	0.57	0.46	1.00		
Item 9	0.46	0.48	0.21	0.32	0.36	0.45	0.61	0.49	1.00	
Item 10	0.47	0.42	0.38	0.47	0.59	0.62	0.60	0.58	0.54	1.00

**Table 4:** Item-to-total statistics by dimension (*Commitment*’s and *Efficacy*’s items) of the *Réceptivité organisationnelle à l’implantation d’un changement*

*ROIC* items	Scale mean if item deleted	Scale variance if item deleted	Corrected item-total Correlation	Squared multiple Correlation	Cronbach’s Alpha if item deleted
Commitment items					
Item 1	12.70	8.97	0.53	0.34	0.84
Item 2	12.78	8.41	0.57	0.40	0.83
Item 3	12.97	7.89	0.62	0.47	0.82
Item 4	13.13	7.29	0.75	0.60	0.78
Item 5	13.00	7.19	0.76	0.61	0.78
Efficacy items					
Item 6	12.21	9.89	0.70	0.53	0.83
Item 7	12.25	9.62	0.71	0.56	0.82
Item 8	12.38	9.83	0.64	0.44	0.84
Item 9	12.93	9.74	0.63	0.44	0.84
Item 10	12.64	9.02	0.72	0.53	0.82

**Table 5:** Item-to-total statistics of the *Réceptivité organisationnelle à l’implantation d’un changement*

*ROIC* items	Scale mean if item deleted	Scale variance if item deleted	Corrected item-total Correlation	Squared multiple Correlation	Cronbach’s Alpha if item deleted
Item 1	28.30	39.56	0.62	0.47	0.90
Item 2	28.38	38.66	0.63	0.46	0.90
Item 3	28.57	38.89	0.54	0.48	0.91
Item 4	28.73	37.12	0.71	0.64	0.90
Item 5	28.60	36.32	0.77	0.70	0.89
Item 6	28.35	36.94	0.78	0.66	0.89
Item 7	28.39	37.14	0.72	0.64	0.90
Item 8	28.52	37.51	0.65	0.49	0.90
Item 9	29.07	38.13	0.58	0.47	0.90
Item 10	28.78	36.27	0.70	0.55	0.90

#### Construct validity of the *ROIC*’s items

The confirmatory factor analysis with the *ROIC*’s results suggested a moderate fit of the French version with the theoretical structure of the ORIC (see Table 6). Four items (2 to 5) intended to document the *Commitment* subscale exhibited factor loadings superior to 0.60, and all five items related to the *Efficacy* subscale reached this threshold. The CFI was equal to 0.89 and the TLI to 0.85, whereas SRMR and RMSEA coefficients reached 0.08 and 0.12 respectively.

**Table 6:** Confirmatory factor analysis of the “Organizational Readiness for Implementing Change” and the *Réceptivité organisationnelle à l’implantation d’un changement*

	Component I		Component II	
	Standardized factor loadings	Standard error	Standardized factor loadings	Standard error
ORIC items (Shea et al., 2014)				
Item 1	0.872	0.025		
Item 2	0.784	0.036		
Item 3	0.769	0.038		
Item 4	0.898	0.021		
Item 5	0.874	0.024		
Item 6			0.838	0.033
Item 7			0.684	0.051
Item 8			0.768	0.041
Item 9			0.800	0.038
Item 10			0.763	0.042
*ROIC* items				
Item 1	0.594	0.075		
Item 2	0.632	0.063		
Item 3	0.677	0.065		
Item 4	0.820	0.042		
Item 5	0.869	0.033		
Item 6			0.838	0.030
Item 7			0.759	0.047
Item 8			0.704	0.097
Item 9			0.625	0.061
Item 10			0.768	0.045
Adjustment measures				
		ORIC	*ROIC*	
Comparative fit index		0.981	0.885	
Tucker-Lewis index		0.975	0.848	
Root mean square residual		0.042	0.079	
Root mean square error of approximation		0.06	0.118	

## Discussion

The purpose of this study was to conduct a French cross-cultural adaptation of the ORIC, a questionnaire which aims to document the organizational readiness of members to implement change within an organization. This cross-cultural adaptation process has resulted in a new French version of the ORIC, the *ROIC*. Preliminary psychometric testing suggests that the *ROIC* has the potential to contribute to the measurement of the construct of organizational readiness in the context of health services by documenting the perceptions of members in an organization about a KT process.

In the French scientific literature, although the importance of readiness among individuals of an organization is also recognized as a prerequisite to implementing change [33], concepts that address organizational readiness are defined differently (see for instance [34, 35]). It is therefore important to situate the construct of organizational readiness in KT studies according to the conceptualization used to illustrate the process. In this study, we mainly used the *ROIC* to document the perceptions of healthcare professionals about their working environment in homecare services, with regards to the component *Recipients* (i.e., individuals involved in the KT process) of the i-*PARIHS* operational model [11]. However, the questionnaire could also inform different levels of context (e.g., local, organizational, external), as suggested in the conceptual frameworks *Promoting Action on Research Implementation in Health Sciences* [36] and *Consolidated Framework for Implementation Research* [37]. To this end, with the use of the ORIC, Sharma et al. (2018) demonstrated that readiness levels varied according to the contextual level studied, which supports the conceptualization of organizational readiness as a multilevel construct [38].

Regarding the reliability of the *ROIC*, when the items were divided into their respective subscales, Cronbach’s alpha values (*Commitment* subscale: α = 0.84 and *Efficacy* subscale: α = 0.86) were slightly inferior to the coefficients obtained by Shea et al. (2014) in the English original version (*Commitment* subscale: α = 0.92 and *Efficacy* subscale: α = 0.88), but remained in the accepted range of homogeneity according to DeVellis (2003) [29]. In this study, the average inter-item correlation coefficients (0.50) and the range of inter-item correlation coefficients [0.21–0.73] were also acceptable (i.e., between 0.15 and 0.50).These results provide initial evidence that the *ROIC*’s items are related to each other and can be used to document the construct of organizational readiness. However, the high values of inter-item correlation coefficients (> 0.50) observed for items 5, 6 and 7 suggest possible redundancy of content. Considering that items 6 and 7 are related to the *Efficacy* subscale, the assumption that the content of these items could be in line with items 6 to 10 is plausible since it refers to the same subscale (see Table 3). Concerning the item-to-total scale correlations, Cronbach’s alpha coefficients (*Commitment* subscale: 0.78 to 0.84; *Efficacy* subscale: 0.82 to 0.84) were lower than the global internal consistency calculated for each subscale (i.e., *Commitment* subscale: α = 0.84; *Efficacy* subscale: α = 0.86). This highlights the importance of each item’s contribution in documenting the subscale to which they refer. While the two facets of the concept of organizational readiness are considered independent, they were still interrelated [5, 14]. This theoretical perspective about the overlap of concepts through the two subscales of the *ROIC* can also be illustrated with the item-to-total scale statistics of the 10-item version, with Cronbach’s alpha coefficients slightly varying between 0.89 and 0.91, which denotes the tool’s stable internal consistency.

Initial documentation of the *ROIC*’s model fit using confirmatory factor analysis suggests that the French version of the ORIC’s items can distinguish the two initial subscales of the ORIC’s original version, and provides initial evidence for the *ROIC*’s construct validity. However, this statement should be nuanced for certain items of the French version of the questionnaire and illustrates the challenge of operationalizing the initial conceptualization of organizational readiness’ components view theoretically as independent factors. Indeed, the factor loading of item 1 is slightly inferior to 0.60 - lower than that obtained in Shea et al.’s initial confirmatory factor analysis [5]. This observation can be triangulated with the lower squared multiple correlation noted for item 1, as it tends to explain a lower proportion of variance than the other items of the *Commitment* subscale (see Table 4). Although they are near the threshold initially established, the factor loadings of items 2, 3 and 9 are also lower than the ones obtained in the original version of the ORIC (see Table 6). Moreover, the values of the CFI (0.89) and the TLI (0.85) are slightly inferior to the pre-established threshold (≥ 0.95), and the value of the RMSEA (0.12) is slightly superior to the allowed error value (< 0.06) according to Hu & Bentler (1999) [32]. Although the sample size (*n* = 125) of this study could be considered sufficient according to the rule of thumb using the number of variables (*N*/*p* [number of variables] ≥ 10), it does not meet the two other rules usually used in confirmatory factor analysis (i.e., number of parameters [N/q ≥ 5] and sample size [*n* ≥ 200]) [39, 40]. While the CFI and TLI indexes are less sensitive to sample size than SRMR and RMSEA indexes, the TLI and RMSEA indexes tend to reject the true population model when the sample size is small [32, 41, 42]. Even though these adjustment measures are useful to indicate that equivalence between the *ROIC’*s and the ORIC’s models may not yet be satisfactory according to some items and the sample size, their original theory-testing purpose should be emphasized. In this perspective, results from the *ROIC*’s confirmatory factor analysis could also suggest that linguistic validation allowed for selecting the optimal French terms corresponding partly to the original item’s underlying concepts. Storkholm et al. (2018) experienced similar issues with vocabulary (e.g., signification of the term “investment”) when translating and testing the ORIC into a Danish version, and they elected to use an 11-item version presenting the optimal fit [43]. Seeing as several conceptual differences were identified during the cross-cultural adaptation process (see Table 1), it may be that the cultural equivalence between French and English concepts in the field of organizational change, as they appear in the ORIC, is imperfect. For instance, item 1, which shows a relatively weak association with its subscale (*Commitment*), includes the notion of “being committed”, which can be translated into either “s’engager” or “s’investir” depending on the context.

### Limits

This study has some limitations. First, contrary to the recommendations of Beaton et al. (2000) concerning the translators’ different backgrounds with regards to the field covered by the questionnaire (i.e., soliciting an expert translator and a “naive” translator), translators Forward1 and Forward2 (step 1) were both “naive” to the field of the ORIC. Moreover, the pretest of the cross-cultural adaptation process completed at step 5 by occupational therapists was not followed by interviews with each respondent to document their perspective on the meaning of each item. However, the pre-final version of the *ROIC* was tested with 9 participants (i.e., 2 homecare managers and 7 occupational therapists) using the “think aloud technique”, and respondents (2 out of 125) who had some difficulties with items of the *ROIC* had the opportunity to clarify their uncertainties in a “Comments” area. Secondly, given that the professional regulatory board organization does not allow for a reminder e-mail to participants who previously complete the questionnaire, the test-retest reliability could not be calculated in this study. As the occupational therapists only completed the *ROIC* (and not the English version of the ORIC), a French validation of the new version was not done as a last step in the cross-cultural process. Thirdly, contrary to the recommendations of Vallerand (1989), the translators who participated in steps 1 and 3 of this cross-cultural adaptation process, as well as the author of the original version of the questionnaire, did not participate in the expert review (step 4) because of availability issues. However, in order to minimize researcher bias (e.g., risk of misunderstanding the terms of the original version as used by the authors), their comments collected throughout the process were carefully considered and used for step 4. Finally, the participants’ sociodemographic characteristics were compared with Quebec’s population of occupational therapists in general, because data pertaining to the population of occupational therapists specifically working within homecare services was unavailable (step 5). However, seeing as occupational therapists from distinct administrative regions of Quebec do not have considerable linguistic cultural differences, these characteristics should not have significant implications for the cross-cultural adaptation process conducted.

### Strengths

The first strength of the study is that it complies with the protocol described by Beaton et al. (2000). The use of Massoubre’s taxonomy to document different types of equivalence also helped perform an in-depth analysis of the variations in the terms chosen during the meeting with the expert committee (step 4). Finally, the absence of missing data despite a considerable sample size (125 respondents) for step 5 (*Pretest*) also allowed to conduct a rigorous analysis of the French version of the ORIC.

Future studies will be needed to conduct additional psychometric analyses of the *ROIC*, to document other aspects of its reliability (e.g., test-retest) and to further assess its construct validity (e.g., concurrent and divergent validity with other well-recognized tools in French literature). With regards to the values of the adjustment measures for the current version of the *ROIC*, further translation revisions with the 10-item version as well as confirmatory factor analyses with a pretested 12-item version might help elaborate different models and find the optimal fit with Weiner’ conceptualization of organizational readiness, as per the original version. To this end, it would be particularly relevant to test the ability of the *ROIC* to predict the issue of an organizational change following its implementation. In a broader contextual perspective, the evaluation of the *ROIC*’s usefulness for professionals from various disciplines (e.g., economy) and organizations could also be relevant to expand the scope and relevance of measuring organizational readiness throughout the implementation of a change.

## Conclusions

In a context where the importance of theory-based KT measures is increasingly being advocated for the development and validation of tools with known measurement properties, it is essential to put effort into making existing questionnaires available to researchers and clinicians involved in the planning and implementation of a change [16]. The ORIC, now culturally adapted to the French language (i.e., the *ROIC*), has the potential to support organizations in documenting their member’s readiness for the development of individual- and context-specific interventions. Considering the intra-organizational and dynamic nature of the concept of organizational readiness [44, 45], the use of questionnaires such as the *ROIC* should be contextualized to give a relevant and authentic picture of the organization and orientate its members in the identification of subsequent efforts for implementing a change.

## Additional file

Additional file 1 **Table S1**“Organizational Readiness for Implementing Change” French version: *Réceptivité organisationnelle à l’implantation d’un changement (DOCX 15 kb)*

## Data Availability

The dataset used and analyzed specifically for the descriptive statistics related to the pretest (step 5) is part of a larger study on the knowledge translation process of Algo and is available from the corresponding author upon request. The conclusions of this article related to the cross-cultural adaptation process of the ORIC have been disseminated through an oral presentation at the 84th congress of the Association francophone pour le savoir (ACFAS) and a poster presentation at the symposium of the Société québécoise de psychologie du travail et des organisations (SQPTO) in 2016.

## Abbreviations

CFI: Comparative fit index
i-*PARIHS*: integrated-*Promoting Action on Research Implementation in Health Services*
KT: Knowledge translation
ORIC: Organizational Readiness for Implementing Change
RMSEA: Root mean square error of approximation
*ROIC*: *Réceptivité organisationnelle à l’implantation d’un changement*
SRMR: Standardized root mean square residual
TLI: Tucker-Lewis index
