# Supplementary material for: French cross-cultural adaptation of the Organizational Readiness for Implementing Change (ORIC)
Source: BMC Health Serv Res. 2019 Jul 31;19:535. doi: 10.1186/s12913-019-4361-1 (PMC6668068; doi:10.1186/s12913-019-4361-1)
Supplement: Supplementary file 1 — Table S1“Organizational Readiness for Implementing Change” French version: Réceptivité organisationnelle à l’implantation d’un changement (DOCX 15 kb) [file 12913_2019_4361_MOESM1_ESM.docx]

**Additional file 1 - "Organizational Readiness for Implementing Change" French version:
*Réceptivité organisationnelle à l’implantation d’un changement***

| 1 | 2 | 3 | 4 | | 5 |  |
| --- | --- | --- | --- | --- | --- | --- |
| En désaccord | Plutôt en désaccord | Ni d’accord ni  en désaccord | Plutôt d’accord | | D’accord |  |
| 1. Les personnes qui travaillent ici* s’engagent à mettre en œuvre un changement de pratique clinique. | | | | | 1 2 3 4 5 | |
| 1. Les personnes qui travaillent ici feront tout ce qui est nécessaire pour mettre en œuvre un changement de pratique clinique. | | | | | 1 2 3 4 5 | |
| 1. Les personnes qui travaillent ici veulent mettre en œuvre un changement de pratique clinique. | | | | | 1 2 3 4 5 | |
| 1. Les personnes qui travaillent ici sont déterminées à mettre en œuvre un changement de pratique clinique. | | | | | 1 2 3 4 5 | |
| 1. Les personnes qui travaillent ici sont motivées à mettre en œuvre un changement de pratique clinique. | | | | | 1 2 3 4 5 | |
| 1. Les personnes qui travaillent ici sont confiantes de pouvoir relever d’éventuels défis liés à la mise en œuvre d’un changement de pratique clinique. | | | | | 1 2 3 4 5 | |
| 1. Les personnes qui travaillent ici sont confiantes de pouvoir suivre l’avancement de la mise en œuvre d’un changement de pratique clinique. | | | | | 1 2 3 4 5 | |
| 1. Les personnes qui travaillent ici sont confiantes de pouvoir coordonner les tâches afin que la mise en œuvre d’un changement de pratique clinique se passe bien. | | | | | 1 2 3 4 5 | |
| 1. Les personnes qui travaillent ici sont confiantes que l’organisation peut les soutenir en cours d’adaptation au changement de pratique clinique. | | | | | 1 2 3 4 5 | |
| 1. Les personnes qui travaillent ici sont confiantes de pouvoir gérer les enjeux de pouvoir et de reconnaissance au sein du groupe durant la mise en œuvre d’un changement de pratique clinique. | | | | | 1 2 3 4 5 | |

*« Les personnes qui travaillent ici » réfèrent aux individus qui travaillent dans votre équipe, au sein de votre organisation. »
